# {μ-5-[1,3-Bis(2,4,6-trimethyl­phen­yl)-3*H*-imidazolium-2-yl]-2-(2-oxoethenyl-1κ*C* ^1^)furan-3-yl-2κ*C* ^3^}-μ-hydrido-bis(tetra­carbonyl­rhenium) tetra­hydro­furan 0.67-solvate

**DOI:** 10.1107/S1600536812006587

**Published:** 2012-02-17

**Authors:** Marilé Landman, Belinda van Westhuizen, Daniela I. Bezuidenhout, David C. Liles

**Affiliations:** a Department of Chemistry, University of Pretoria, Private Bag X20, Hatfield 0028, South Africa

## Abstract

The title complex, [Re_2_(C_27_H_25_N_2_O_2_)H(CO)_8_]·0.67C_4_H_8_O, was formed as a product in the reaction of a rhenium(I)–Fischer carbene complex with a free NHC carbene. The coordination environment about the two Re atoms is slightly distorted octahedral, including a bridging H atom. The imidazolium and furan groups are almost coplanar, whereas the mesityl substituents show an almost perpendicular arrangement with respect to both heterocyclic units. Mol­ecules of the complex pack in such a way as to form channels parallel with the *bc* unit-cell face diagonal running through the unit face diagonal. These channels are partially occupied by tetra­hydro­furan solvent mol­ecules.

## Related literature

For other examples of ketenyl complexes, see: Kreissl *et al.* (1976 ▶, 1977 ▶); Li *et al.* (2006 ▶). Recent examples of dimesityl­imidazol-2-yl groups bonded to a C atom have been reported by: Naeem *et al.* (2010 ▶); Chia *et al.* (2011 ▶).
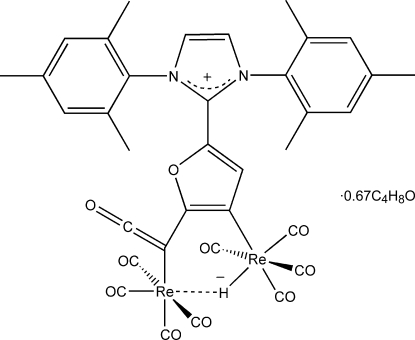


## Experimental

### 

#### Crystal data

[Re_2_(C_27_H_25_N_2_O_2_)H(CO)_8_]·0.67C_4_H_8_O
*M* *_r_* = 1054.99
Triclinic, 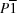

*a* = 12.7058 (7) Å
*b* = 13.8293 (8) Å
*c* = 13.9679 (8) Å
α = 60.760 (1)°
β = 77.680 (1)°
γ = 89.564 (1)°
*V* = 2078.8 (2) Å^3^
*Z* = 2
Mo *K*α radiation
μ = 5.87 mm^−1^
*T* = 293 K
0.27 × 0.18 × 0.06 mm

#### Data collection

Siemens *P*4 diffractometer with SMART 1000 CCD detector
Absorption correction: multi-scan (*SADABS*; Bruker, 2001 ▶) *T*_min_ = 0.366, *T*_max_ = 0.703
11527 measured reflections
7743 independent reflections
6404 reflections with *I* > 2σ(*I*)
*R*_int_ = 0.025

#### Refinement

*R*[*F* ^2^ > 2σ(*F* ^2^)] = 0.042
*wR*(*F* ^2^) = 0.137
*S* = 1.16
7743 reflections
456 parameters
H-atom parameters constrained
Δρ_max_ = 2.10 e Å^−3^
Δρ_min_ = −0.83 e Å^−3^

### 

Data collection: *SMART* (Bruker, 2001 ▶); cell refinement: *SAINT* (Bruker, 2001 ▶); data reduction: *SAINT*; program(s) used to solve structure: *SHELXTL* (Sheldrick, 2008 ▶); program(s) used to refine structure: *SHELXTL* and *SHELXL97* (Sheldrick, 2008 ▶); molecular graphics: *ORTEP-3 for Windows* (Farrugia, 1997 ▶), *POV-RAY* (Cason, 2004 ▶) and *Mercury* (Macrae *et al.*, 2008 ▶); software used to prepare material for publication: *SHELXL97* and *PLATON* (Spek, 2009 ▶).

## Supplementary Material

Crystal structure: contains datablock(s) I, global. DOI: 10.1107/S1600536812006587/im2354sup1.cif

Structure factors: contains datablock(s) I. DOI: 10.1107/S1600536812006587/im2354Isup2.hkl

Additional supplementary materials:  crystallographic information; 3D view; checkCIF report

## Figures and Tables

**Table 1:** r.m.s. deviations of atoms (δ_r.m.s._, Å) and dihedral angles between planes (°) for selected mean planes

Plane	Atoms	δ_r.m.s._	Plane:1	2	3	4
1	C20–C26	0.001	–			
2	C29–C34	0.011	34.8 (4)	–		
3	N1/N2/C15–C17	0.003	84.8 (3)	86.2 (3)	–	
4	C10–C13/O14	0.004	79.3 (5)	87.3 (4)	5.5 (7)	–
5	Re1/Re2/C9–C11	0.041			11.1 (6)	5.7 (4)

**Fig. 1.**
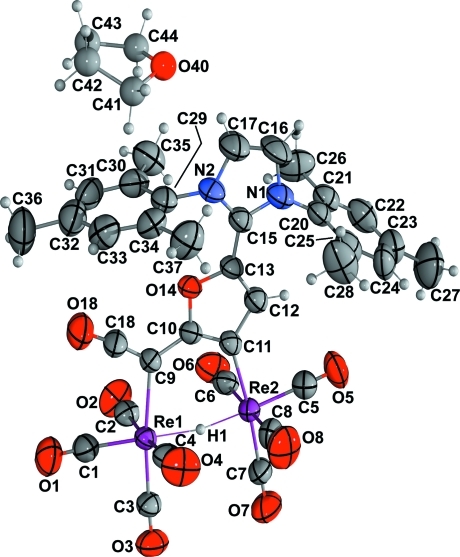
Molecular structure of 1 showing the atomic numbering scheme. Displacement ellipsoids are shown at the 50% probability level. The thf solvent molecule and all H atoms are shown as small spheres of arbitrary radii.

**Fig. 2.**
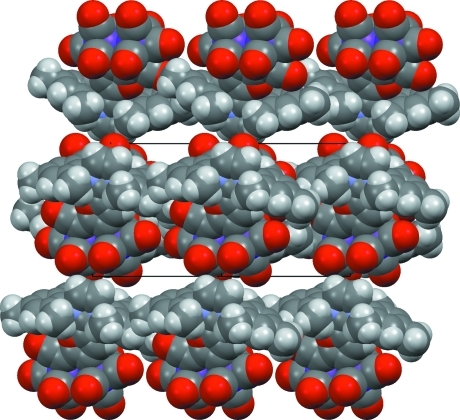
Space-filling model of the crystal structure of 1 with the thf solvent molecules removed. The view is along the b,-c direction showing the channels in the structure running parallel with the b,-c face diagonal.

**Fig. 3.**
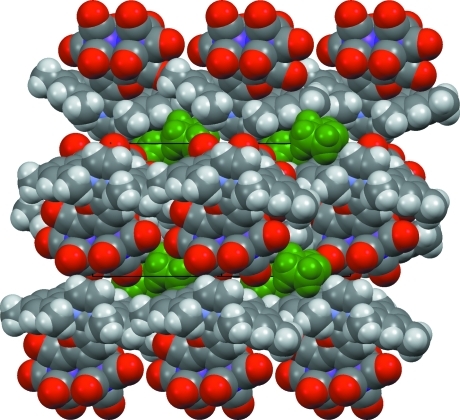
Sspace-filling model of the crystal structure of 1, as in Fig. 2 but with the thf solvent molecules included (shown in green).

## supplementary crystallographic information

### Comment

The title complex (1) was formed as a product in the reaction of a rhenium(I)–Fischer carbene complex with a free NHC carbene, prepared *in situ* by the deprotonation of *N*,*N*'-dimesitylimidazolium chloride (HIMesCl) with *n*-butyllithium. Formation of this complex is quite unique and involves four reaction sites. Rhenium complexes can mimic transition metal Lewis acids to catalyse Friedel–Crafts C—C bond formation. The catalytic activity of the rhenium complexes is initiated by decarbonylation under heating to form the active species [ReBr(CO)_4_]. In complex 1 the position most susceptible to nucleophilic attack is the 2-position on the furan ring. This is attributed to the electron withdrawing effect of the carbene ligand on the ring. Subsequent C—H activation leads to hydride migration and Re—Re bond breaking. The resulting complex now contains both a Re—C and Re—H bond. The Re atom from the original Fischer carbene moiety is left with a vacant coordination site enabling the newly formed hydride to form a bridge between the two Re atoms. Ketenyl complexes of tungsten have been previously reported by Kreissl *et al.* (1976, 1977) and later these types of complexes were also described for rhenium(VII) complexes (Li *et al.*, 2006). The insertion of a bridging CO can lead to the ketene formation observed in complex 1.

The geometry of the dimesitylimidazol-2-yl moiety is similar to those observed for previously published dimesitylimidazol-2-yl structures, in particular for some recent examples bonded to a C atom (Naeem *et al.*, 2010; Chia *et al.*, 2011). As usual, the planes of the rings of the mesityl substituents are close to perpendicular to that of the imidazole ring (Table 1). However, the furan ring is close to being coplanar with the imidazole ring (Table 1).

The overall shape of the molecule of the complex does not lend itself to efficient close-packing in the crystal. The molecules of the complex pack in layers parallel with the *A* face of the unit cell with the two C—H moieties of each imidazol ring in close contact with the Re(CO)_4_ moieties of molecules in the next layer. Such an arrangement leaves voids in the crystal structure which form channels running parallel with the *b*,-*c* face diagonal of the unit cell (Fig. 2). The thf solvent molecules occupy these channels (Fig. 3). The channels allow egress of thf molecules out of the crystal without significant degradation of the structure. Thus loss of solvent has occurred leading to a partial occupancy of the site by the thf solvate of 0.666 (13) of a molecule per asymmetric unit. Uncertainty in the precise positions of the thf molecules within the channels leads to the molecule being ill-defined in the structure solution and refinement, thus the thf molecule needed to be treated as a rigid body.

### Experimental

HIMesCl (1 mmol, 0.34 g) was dissolved in thf and cooled to -78°C. nBuLi (1 mmol, 0.7 ml) was added and stirred for 20 min after which the Fischer carbene, [*eq*Re_2_(CO)_9_{C(OEt)Fu}], (1 mmol, 0.75 g) was added. The solution was stirred at -78°C for 1hr, 30 min at -30°C and then allowed to warm to room temperature. The solvents were removed *in vacuo* and purification was done using cold column chromatography on florasil. The unreacted starting material was eluted with dcm and the polar fraction collected using thf as eluent. Complex 1 was crystallized from a thf solution (yield: 50 mg, 5%).

^1^H NMR (δ, p.p.m.), C_6_D_6_: -15.48 (s, 1H), 2.06 (br, 6H), 2.07 (br, 6H), 2.19 (br, 6H), 6.70 (s, 4H), 7.63 (s, 2H); ^13^C NMR (δ, p.p.m.), C_6_D_6_: 16.9, 20.6, 125.5, 129.3, 130.4, 136.7, 141.9, 147.0, 148.8, 159.2, 164.4, 249.8, 275.4. IR (dcm): ν_CO_ (cm^-1^) 2086, 1970, 1948, 1937, 1932, 1903, 1883, 1876.

### Refinement

All H atoms were included in calculated positions and allowed to ride on the atom to which each is bonded (except for H1 which bridges the two Re atoms the coordinates of which were not varied). The isotropic adp's for each H were set to 1.2 × the equivalent isotropic adp of the atom to which each is bonded (1.5 × for H1). A poorly defined thf solvent molecule was treated as a rigid body and a common isotropic adp was refined for all its non-H atoms. A common site occupation factor for all the atoms of the thf molecule refined to a value of 0.666 (13).

### Crystal data

**Table:**

[Re_2_(C_27_H_25_N_2_O_2_)H(CO)_8_]·0.67C_4_H_8_O	*Z* = 2
*M**_r_* = 1054.99	*F*(000) = 1013
Triclinic, *P*1	*D*_x_ = 1.685 Mg m^−^^3^
Hall symbol: -P 1	Mo *K*α radiation, λ = 0.71073 Å
*a* = 12.7058 (7) Å	Cell parameters from 7068 reflections
*b* = 13.8293 (8) Å	θ = 2.7–26.4°
*c* = 13.9679 (8) Å	µ = 5.87 mm^−^^1^
α = 60.760 (1)°	*T* = 293 K
β = 77.680 (1)°	Plate, orange
γ = 89.564 (1)°	0.27 × 0.18 × 0.06 mm
*V* = 2078.8 (2) Å^3^	

### Data collection

**Table:**

Siemens P4 with SMART 1000 CCD detector diffractometer	7743 independent reflections
Radiation source: fine-focus sealed tube	6404 reflections with *I* > 2σ(*I*)
Graphite monochromator	*R*_int_ = 0.025
Detector resolution: 8.3 pixels mm^-1^	θ_max_ = 26.5°, θ_min_ = 2.1°
φ and ω scans	*h* = −15→10
Absorption correction: multi-scan (*SADABS*; Bruker, 2001)	*k* = −14→16
*T*_min_ = 0.366, *T*_max_ = 0.703	*l* = −16→15
11527 measured reflections	

### Refinement

**Table:**

Refinement on *F*^2^	Primary atom site location: structure-invariant direct methods
Least-squares matrix: full	Secondary atom site location: difference Fourier map
*R*[*F*^2^ > 2σ(*F*^2^)] = 0.042	Hydrogen site location: inferred from neighbouring sites
*wR*(*F*^2^) = 0.137	H-atom parameters constrained
*S* = 1.16	*w* = 1/[σ^2^(*F*_o_^2^) + (0.080*P*)^2^ + 2.0563*P*] where *P* = (*F*_o_^2^ + 2*F*_c_^2^)/3
7743 reflections	(Δ/σ)_max_ = 0.002
456 parameters	Δρ_max_ = 2.10 e Å^−^^3^
0 restraints	Δρ_min_ = −0.83 e Å^−^^3^

### Special details

**Table:**

Geometry. All e.s.d.'s (except the e.s.d. in the dihedral angle between two l.s. planes) are estimated using the full covariance matrix. The cell e.s.d.'s are taken into account individually in the estimation of e.s.d.'s in distances, angles and torsion angles; correlations between e.s.d.'s in cell parameters are only used when they are defined by crystal symmetry. An approximate (isotropic) treatment of cell e.s.d.'s is used for estimating e.s.d.'s involving l.s. planes.
Refinement. Refinement of *F*^2^ against ALL reflections. The weighted *R*-factor *wR* and goodness of fit *S* are based on *F*^2^, conventional *R*-factors *R* are based on *F*, with *F* set to zero for negative *F*^2^. The threshold expression of *F*^2^ > σ(*F*^2^) is used only for calculating *R*-factors(gt) *etc*. and is not relevant to the choice of reflections for refinement. *R*-factors based on *F*^2^ are statistically about twice as large as those based on *F*, and *R*-factors based on ALL data will be even larger.

### Fractional atomic coordinates and isotropic or equivalent isotropic displacement parameters (Å^2^)

**Table:**

	*x*	*y*	*z*	*U*_iso_*/*U*_eq_	Occ. (<1)
Re1	0.75162 (2)	1.01206 (2)	0.10173 (2)	0.04870 (12)	
Re2	0.74307 (2)	0.78055 (2)	0.06999 (2)	0.05348 (12)	
H1	0.7648	0.8944	0.0764	0.080*	
C1	0.7453 (7)	1.1333 (7)	0.1313 (7)	0.071 (2)	
O1	0.7459 (7)	1.2100 (7)	0.1450 (7)	0.105 (2)	
C2	0.8343 (6)	0.9350 (7)	0.2195 (7)	0.0610 (18)	
O2	0.8773 (7)	0.8918 (7)	0.2893 (7)	0.100 (2)	
C3	0.8808 (6)	1.0869 (7)	−0.0174 (7)	0.0629 (19)	
O3	0.9567 (6)	1.1295 (7)	−0.0894 (6)	0.095 (2)	
C4	0.6540 (7)	1.0767 (7)	−0.0043 (7)	0.067 (2)	
O4	0.5986 (6)	1.1107 (7)	−0.0618 (7)	0.101 (2)	
C5	0.7160 (8)	0.6440 (8)	0.0693 (8)	0.075 (2)	
O5	0.7008 (7)	0.5623 (6)	0.0714 (7)	0.107 (3)	
C6	0.8170 (8)	0.7038 (7)	0.1952 (7)	0.068 (2)	
O6	0.8582 (7)	0.6603 (7)	0.2671 (7)	0.104 (2)	
C7	0.8785 (7)	0.8279 (8)	−0.0455 (8)	0.073 (2)	
O7	0.9577 (6)	0.8573 (8)	−0.1145 (7)	0.109 (3)	
C8	0.6567 (8)	0.8596 (8)	−0.0450 (8)	0.075 (2)	
O8	0.6070 (8)	0.9024 (8)	−0.1087 (7)	0.113 (3)	
C9	0.6010 (5)	0.9122 (6)	0.2305 (6)	0.0502 (15)	
C10	0.5530 (5)	0.8113 (5)	0.2430 (5)	0.0501 (15)	
C11	0.5923 (6)	0.7459 (6)	0.1963 (6)	0.0529 (16)	
C12	0.5051 (6)	0.6574 (6)	0.2443 (6)	0.0585 (17)	
H12	0.5050	0.5982	0.2303	0.070*	
C13	0.4233 (5)	0.6738 (6)	0.3127 (6)	0.0516 (15)	
O14	0.4522 (4)	0.7695 (4)	0.3141 (4)	0.0531 (11)	
N1	0.2703 (5)	0.5301 (6)	0.3776 (6)	0.0661 (17)	
N2	0.2422 (5)	0.6485 (6)	0.4372 (6)	0.0646 (16)	
C15	0.3163 (6)	0.6195 (6)	0.3746 (6)	0.0556 (16)	
C16	0.1654 (7)	0.5030 (8)	0.4448 (9)	0.086 (3)	
H16	0.1162	0.4443	0.4616	0.104*	
C17	0.1478 (7)	0.5758 (8)	0.4809 (9)	0.084 (3)	
H17	0.0840	0.5778	0.5270	0.101*	
C18	0.5526 (6)	0.9585 (8)	0.2842 (8)	0.067 (2)	
O18	0.5148 (6)	1.0090 (6)	0.3294 (7)	0.105 (2)	
C20	0.3203 (6)	0.4712 (6)	0.3226 (7)	0.0592 (17)	
C21	0.3690 (7)	0.3778 (7)	0.3837 (7)	0.068 (2)	
C22	0.4181 (8)	0.3233 (7)	0.3273 (9)	0.088 (3)	
H22	0.4515	0.2602	0.3659	0.106*	
C23	0.4185 (9)	0.3602 (9)	0.2152 (10)	0.091 (3)	
C24	0.3701 (9)	0.4516 (8)	0.1600 (9)	0.091 (3)	
H24	0.3705	0.4767	0.0848	0.109*	
C25	0.3205 (8)	0.5084 (7)	0.2099 (8)	0.077 (2)	
C26	0.3714 (10)	0.3376 (10)	0.5035 (8)	0.106 (4)	
H26A	0.4163	0.2780	0.5277	0.159*	
H26B	0.2992	0.3112	0.5505	0.159*	
H26C	0.4004	0.3978	0.5095	0.159*	
C27	0.4768 (15)	0.2985 (13)	0.1582 (14)	0.153 (6)	
H27A	0.4400	0.3016	0.1036	0.230*	
H27B	0.4771	0.2220	0.2141	0.230*	
H27C	0.5501	0.3328	0.1210	0.230*	
C28	0.2608 (11)	0.6106 (8)	0.1472 (10)	0.112 (4)	
H28A	0.2673	0.6256	0.0713	0.169*	
H28B	0.2926	0.6743	0.1455	0.169*	
H28C	0.1856	0.5951	0.1860	0.169*	
C29	0.2527 (6)	0.7402 (7)	0.4599 (7)	0.0638 (19)	
C30	0.2917 (7)	0.7179 (8)	0.5537 (7)	0.072 (2)	
C31	0.3037 (8)	0.8072 (9)	0.5709 (8)	0.084 (3)	
H31	0.3320	0.7960	0.6313	0.100*	
C32	0.2764 (9)	0.9113 (10)	0.5041 (10)	0.091 (3)	
C33	0.2344 (8)	0.9257 (8)	0.4147 (8)	0.085 (3)	
H33	0.2141	0.9953	0.3692	0.102*	
C34	0.2210 (7)	0.8417 (8)	0.3895 (7)	0.072 (2)	
C35	0.3235 (9)	0.6052 (8)	0.6298 (9)	0.099 (3)	
H35A	0.2615	0.5502	0.6632	0.148*	
H35B	0.3495	0.6078	0.6883	0.148*	
H35C	0.3797	0.5859	0.5862	0.148*	
C36	0.2952 (12)	1.0079 (10)	0.5234 (12)	0.127 (5)	
H36A	0.3062	1.0767	0.4530	0.191*	
H36B	0.3582	0.9997	0.5532	0.191*	
H36C	0.2332	1.0084	0.5761	0.191*	
C37	0.1771 (9)	0.8593 (9)	0.2922 (8)	0.095 (3)	
H37A	0.2336	0.8561	0.2362	0.142*	
H37B	0.1507	0.9309	0.2599	0.142*	
H37C	0.1189	0.8021	0.3179	0.142*	
O40	−0.0474 (14)	0.6479 (15)	0.6302 (16)	0.198 (6)*	0.666 (13)
C41	−0.0517 (17)	0.7624 (15)	0.5829 (14)	0.198 (6)*	0.666 (13)
H41A	−0.1062	0.7858	0.5391	0.237*	0.666 (13)
H41B	0.0179	0.8029	0.5338	0.237*	0.666 (13)
C42	−0.0803 (17)	0.7828 (14)	0.679 (2)	0.198 (6)*	0.666 (13)
H42A	−0.0493	0.8563	0.6585	0.237*	0.666 (13)
H42B	−0.1584	0.7766	0.7058	0.237*	0.666 (13)
C43	−0.0338 (19)	0.6956 (19)	0.7661 (15)	0.198 (6)*	0.666 (13)
H43A	−0.0846	0.6624	0.8395	0.237*	0.666 (13)
H43B	0.0325	0.7257	0.7708	0.237*	0.666 (13)
C44	−0.0114 (17)	0.6130 (13)	0.7289 (16)	0.198 (6)*	0.666 (13)
H44A	0.0658	0.6069	0.7143	0.237*	0.666 (13)
H44B	−0.0487	0.5405	0.7874	0.237*	0.666 (13)

### Atomic displacement parameters (Å^2^)

**Table:**

	*U*^11^	*U*^22^	*U*^33^	*U*^12^	*U*^13^	*U*^23^
Re1	0.04431 (17)	0.05048 (18)	0.04742 (18)	−0.00269 (12)	−0.00936 (12)	−0.02200 (14)
Re2	0.05102 (19)	0.0588 (2)	0.05106 (19)	−0.00255 (13)	−0.00578 (13)	−0.03011 (15)
C1	0.076 (5)	0.063 (5)	0.068 (5)	0.005 (4)	−0.004 (4)	−0.032 (4)
O1	0.131 (7)	0.094 (5)	0.108 (6)	0.025 (5)	−0.023 (5)	−0.067 (5)
C2	0.047 (4)	0.073 (5)	0.061 (5)	0.004 (4)	−0.015 (4)	−0.032 (4)
O2	0.095 (5)	0.131 (6)	0.087 (5)	0.041 (5)	−0.050 (4)	−0.054 (5)
C3	0.054 (4)	0.074 (5)	0.057 (4)	−0.010 (4)	−0.008 (4)	−0.031 (4)
O3	0.076 (4)	0.117 (6)	0.070 (4)	−0.025 (4)	0.003 (3)	−0.037 (4)
C4	0.058 (5)	0.067 (5)	0.059 (5)	−0.001 (4)	−0.018 (4)	−0.018 (4)
O4	0.085 (5)	0.110 (6)	0.096 (5)	0.026 (4)	−0.049 (4)	−0.033 (4)
C5	0.077 (6)	0.077 (6)	0.071 (5)	−0.002 (4)	−0.011 (4)	−0.041 (5)
O5	0.146 (7)	0.069 (4)	0.120 (6)	−0.007 (4)	−0.041 (5)	−0.054 (4)
C6	0.074 (5)	0.058 (5)	0.056 (5)	−0.004 (4)	−0.012 (4)	−0.017 (4)
O6	0.109 (6)	0.094 (5)	0.082 (5)	0.007 (4)	−0.039 (5)	−0.017 (4)
C7	0.068 (5)	0.087 (6)	0.068 (5)	−0.009 (4)	0.000 (4)	−0.048 (5)
O7	0.082 (5)	0.148 (7)	0.099 (5)	−0.013 (5)	0.017 (4)	−0.078 (5)
C8	0.088 (6)	0.078 (6)	0.068 (5)	0.001 (5)	−0.022 (5)	−0.041 (5)
O8	0.141 (8)	0.123 (7)	0.094 (5)	0.033 (6)	−0.068 (6)	−0.053 (5)
C9	0.047 (4)	0.053 (4)	0.052 (4)	−0.001 (3)	−0.009 (3)	−0.028 (3)
C10	0.049 (4)	0.049 (4)	0.045 (3)	−0.002 (3)	−0.011 (3)	−0.018 (3)
C11	0.047 (4)	0.056 (4)	0.053 (4)	−0.003 (3)	−0.015 (3)	−0.025 (3)
C12	0.058 (4)	0.057 (4)	0.059 (4)	−0.001 (3)	−0.015 (3)	−0.027 (4)
C13	0.041 (3)	0.055 (4)	0.053 (4)	−0.006 (3)	−0.007 (3)	−0.024 (3)
O14	0.043 (2)	0.057 (3)	0.051 (3)	−0.006 (2)	−0.004 (2)	−0.024 (2)
N1	0.051 (4)	0.067 (4)	0.071 (4)	−0.008 (3)	−0.010 (3)	−0.029 (3)
N2	0.043 (3)	0.075 (4)	0.070 (4)	−0.012 (3)	−0.003 (3)	−0.036 (4)
C15	0.052 (4)	0.055 (4)	0.055 (4)	−0.004 (3)	−0.015 (3)	−0.024 (3)
C16	0.051 (5)	0.087 (6)	0.108 (7)	−0.025 (4)	−0.005 (5)	−0.044 (6)
C17	0.055 (5)	0.091 (6)	0.101 (7)	−0.011 (4)	−0.003 (5)	−0.050 (6)
C18	0.052 (4)	0.082 (5)	0.075 (5)	−0.008 (4)	−0.001 (4)	−0.050 (5)
O18	0.098 (5)	0.113 (5)	0.126 (6)	−0.002 (4)	0.002 (4)	−0.087 (5)
C20	0.056 (4)	0.053 (4)	0.063 (4)	−0.009 (3)	−0.015 (3)	−0.024 (4)
C21	0.065 (5)	0.062 (5)	0.072 (5)	−0.005 (4)	−0.023 (4)	−0.026 (4)
C22	0.092 (7)	0.058 (5)	0.109 (8)	0.011 (5)	−0.039 (6)	−0.031 (5)
C23	0.102 (8)	0.083 (6)	0.101 (8)	0.009 (6)	−0.025 (6)	−0.056 (6)
C24	0.132 (9)	0.080 (6)	0.080 (6)	−0.005 (6)	−0.040 (6)	−0.048 (5)
C25	0.087 (6)	0.061 (5)	0.083 (6)	−0.007 (4)	−0.039 (5)	−0.029 (4)
C26	0.110 (9)	0.110 (8)	0.066 (6)	0.017 (7)	−0.028 (6)	−0.016 (6)
C27	0.204 (17)	0.129 (11)	0.168 (14)	0.023 (11)	−0.031 (12)	−0.110 (11)
C28	0.158 (11)	0.069 (6)	0.110 (9)	0.021 (6)	−0.087 (9)	−0.022 (6)
C29	0.053 (4)	0.067 (5)	0.070 (5)	−0.003 (3)	−0.005 (4)	−0.036 (4)
C30	0.061 (5)	0.086 (6)	0.060 (5)	0.001 (4)	−0.016 (4)	−0.029 (4)
C31	0.081 (6)	0.111 (8)	0.069 (5)	0.003 (5)	−0.017 (5)	−0.054 (6)
C32	0.091 (7)	0.105 (8)	0.097 (7)	0.013 (6)	−0.018 (6)	−0.067 (7)
C33	0.088 (6)	0.083 (6)	0.079 (6)	0.018 (5)	−0.018 (5)	−0.038 (5)
C34	0.067 (5)	0.081 (6)	0.064 (5)	0.012 (4)	−0.012 (4)	−0.036 (5)
C35	0.103 (8)	0.089 (7)	0.091 (7)	−0.004 (6)	−0.039 (6)	−0.029 (6)
C36	0.170 (13)	0.113 (9)	0.143 (12)	0.027 (9)	−0.045 (10)	−0.094 (9)
C37	0.110 (8)	0.098 (7)	0.079 (6)	0.029 (6)	−0.039 (6)	−0.040 (6)

### Geometric parameters (Å, º)

**Table:**

Re1—C1	1.909 (9)	C23—C27	1.521 (16)
Re1—C3	1.926 (8)	C24—C25	1.356 (14)
Re1—C2	1.989 (8)	C24—H24	0.93
Re1—C4	2.001 (8)	C25—C28	1.548 (12)
Re1—C9	2.225 (7)	C26—H26A	0.96
Re1—H1	1.83	C26—H26B	0.96
Re2—C5	1.927 (9)	C26—H26C	0.96
Re2—C7	1.947 (9)	C27—H27A	0.96
Re2—C6	1.984 (9)	C27—H27B	0.96
Re2—C8	1.999 (10)	C27—H27C	0.96
Re2—C11	2.182 (7)	C28—H28A	0.96
Re2—H1	1.65	C28—H28B	0.96
C1—O1	1.167 (10)	C28—H28C	0.96
C2—O2	1.121 (10)	C29—C34	1.383 (12)
C3—O3	1.142 (10)	C29—C30	1.393 (11)
C4—O4	1.108 (10)	C30—C31	1.384 (13)
C5—O5	1.133 (10)	C30—C35	1.508 (13)
C6—O6	1.129 (11)	C31—C32	1.372 (14)
C7—O7	1.147 (11)	C31—H31	0.9300
C8—O8	1.123 (12)	C32—C33	1.387 (14)
C9—C18	1.272 (10)	C32—C36	1.515 (14)
C9—C10	1.442 (9)	C33—C34	1.388 (13)
C10—O14	1.366 (8)	C33—H33	0.93
C10—C11	1.387 (10)	C34—C37	1.488 (12)
C11—C12	1.441 (10)	C35—H35A	0.96
C12—C13	1.353 (10)	C35—H35B	0.96
C12—H12	0.93	C35—H35C	0.96
C13—O14	1.386 (8)	C36—H36A	0.96
C13—C15	1.429 (9)	C36—H36B	0.96
N1—C15	1.349 (10)	C36—H36C	0.96
N1—C16	1.389 (11)	C37—H37A	0.96
N1—C20	1.433 (10)	C37—H37B	0.96
N2—C15	1.340 (10)	C37—H37C	0.96
N2—C17	1.388 (10)	O40—C41	1.39
N2—C29	1.463 (11)	O40—C44	1.40
C16—C17	1.328 (13)	C41—C42	1.48
C16—H16	0.93	C41—H41A	0.97
C17—H17	0.93	C41—H41B	0.97
C18—O18	1.188 (10)	C42—C43	1.46
C20—C21	1.384 (11)	C42—H42A	0.97
C20—C25	1.397 (11)	C42—H42B	0.97
C21—C22	1.394 (13)	C43—C44	1.47
C21—C26	1.492 (13)	C43—H43A	0.97
C22—C23	1.388 (14)	C43—H43B	0.97
C22—H22	0.93	C44—H44A	0.97
C23—C24	1.345 (14)	C44—H44B	0.97
			
C1—Re1—C3	90.7 (4)	C25—C24—H24	118.7
C1—Re1—C2	89.7 (4)	C24—C25—C20	118.9 (8)
C3—Re1—C2	93.3 (3)	C24—C25—C28	122.1 (9)
C1—Re1—C4	92.2 (4)	C20—C25—C28	119.0 (9)
C3—Re1—C4	93.0 (3)	C21—C26—H26A	109.5
C2—Re1—C4	173.4 (3)	C21—C26—H26B	109.5
C1—Re1—C9	96.0 (3)	H26A—C26—H26B	109.5
C3—Re1—C9	173.0 (3)	C21—C26—H26C	109.5
C2—Re1—C9	88.6 (3)	H26A—C26—H26C	109.5
C4—Re1—C9	85.0 (3)	H26B—C26—H26C	109.5
C1—Re1—H1	177.0	C23—C27—H27A	109.5
C3—Re1—H1	88.0	C23—C27—H27B	109.5
C2—Re1—H1	87.0	H27A—C27—H27B	109.5
C4—Re1—H1	91.0	C23—C27—H27C	109.5
C9—Re1—H1	85.0	H27A—C27—H27C	109.5
C5—Re2—C7	93.7 (4)	H27B—C27—H27C	109.5
C5—Re2—C6	91.3 (4)	C25—C28—H28A	109.5
C7—Re2—C6	92.9 (4)	C25—C28—H28B	109.5
C5—Re2—C8	90.9 (4)	H28A—C28—H28B	109.5
C7—Re2—C8	92.4 (4)	C25—C28—H28C	109.5
C6—Re2—C8	174.2 (4)	H28A—C28—H28C	109.5
C5—Re2—C11	92.4 (3)	H28B—C28—H28C	109.5
C7—Re2—C11	173.8 (3)	C34—C29—C30	124.2 (8)
C6—Re2—C11	88.4 (3)	C34—C29—N2	118.5 (8)
C8—Re2—C11	86.1 (3)	C30—C29—N2	117.2 (8)
C5—Re2—H1	178.0	C31—C30—C29	115.7 (8)
C7—Re2—H1	88.0	C31—C30—C35	121.6 (8)
C6—Re2—H1	87.0	C29—C30—C35	122.6 (9)
C8—Re2—H1	91.0	C32—C31—C30	123.8 (9)
C11—Re2—H1	86.0	C32—C31—H31	118.1
O1—C1—Re1	176.7 (8)	C30—C31—H31	118.1
O2—C2—Re1	176.9 (8)	C33—C32—C31	116.9 (9)
O3—C3—Re1	178.0 (8)	C33—C32—C36	121.1 (11)
O4—C4—Re1	178.7 (9)	C31—C32—C36	121.9 (10)
O5—C5—Re2	178.3 (9)	C32—C33—C34	123.5 (9)
O6—C6—Re2	179.3 (10)	C32—C33—H33	118.2
O7—C7—Re2	178.9 (11)	C34—C33—H33	118.2
O8—C8—Re2	178.8 (10)	C29—C34—C33	115.7 (8)
C18—C9—C10	121.6 (7)	C29—C34—C37	121.6 (9)
C18—C9—Re1	114.0 (6)	C33—C34—C37	122.7 (9)
C10—C9—Re1	123.8 (5)	C30—C35—H35A	109.5
O14—C10—C11	112.6 (6)	C30—C35—H35B	109.5
O14—C10—C9	116.3 (6)	H35A—C35—H35B	109.5
C11—C10—C9	131.1 (6)	C30—C35—H35C	109.5
C10—C11—C12	102.7 (6)	H35A—C35—H35C	109.5
C10—C11—Re2	127.3 (5)	H35B—C35—H35C	109.5
C12—C11—Re2	129.7 (5)	C32—C36—H36A	109.5
C13—C12—C11	109.4 (7)	C32—C36—H36B	109.5
C13—C12—H12	125.3	H36A—C36—H36B	109.5
C11—C12—H12	125.3	C32—C36—H36C	109.5
C12—C13—O14	109.3 (6)	H36A—C36—H36C	109.5
C12—C13—C15	134.2 (7)	H36B—C36—H36C	109.5
O14—C13—C15	116.4 (6)	C34—C37—H37A	109.5
C10—O14—C13	106.0 (5)	C34—C37—H37B	109.5
C15—N1—C16	108.4 (7)	H37A—C37—H37B	109.5
C15—N1—C20	126.5 (7)	C34—C37—H37C	109.5
C16—N1—C20	125.1 (7)	H37A—C37—H37C	109.5
C15—N2—C17	109.1 (7)	H37B—C37—H37C	109.5
C15—N2—C29	128.3 (6)	C41—O40—C44	106.5
C17—N2—C29	122.6 (7)	O40—C41—C42	105.7
N2—C15—N1	107.3 (6)	O40—C41—H41A	110.6
N2—C15—C13	127.2 (7)	C42—C41—H41A	110.6
N1—C15—C13	125.5 (7)	O40—C41—H41B	110.6
C17—C16—N1	107.9 (8)	C42—C41—H41B	110.6
C17—C16—H16	126.1	H41A—C41—H41B	108.7
N1—C16—H16	126.1	C43—C42—C41	104.2
C16—C17—N2	107.3 (8)	C43—C42—H42A	110.9
C16—C17—H17	126.4	C41—C42—H42A	110.9
N2—C17—H17	126.4	C43—C42—H42B	110.9
O18—C18—C9	174.3 (9)	C41—C42—H42B	110.9
C21—C20—C25	121.2 (8)	H42A—C42—H42B	108.9
C21—C20—N1	118.3 (7)	C42—C43—C44	104.7
C25—C20—N1	120.4 (7)	C42—C43—H43A	110.8
C20—C21—C22	116.8 (8)	C44—C43—H43A	110.8
C20—C21—C26	122.2 (9)	C42—C43—H43B	110.8
C22—C21—C26	121.0 (9)	C44—C43—H43B	110.8
C23—C22—C21	122.0 (9)	H43A—C43—H43B	108.9
C23—C22—H22	119.0	O40—C44—C43	108.5
C21—C22—H22	119.0	O40—C44—H44A	110.0
C24—C23—C22	118.5 (9)	C43—C44—H44A	110.0
C24—C23—C27	122.5 (11)	O40—C44—H44B	110.0
C22—C23—C27	119.0 (11)	C43—C44—H44B	110.0
C23—C24—C25	122.5 (9)	H44A—C44—H44B	108.4
C23—C24—H24	118.7		
			
C1—Re1—C9—C18	1.3 (7)	C15—N1—C20—C21	94.5 (9)
C2—Re1—C9—C18	90.8 (7)	C16—N1—C20—C21	−84.7 (10)
C4—Re1—C9—C18	−90.4 (7)	C15—N1—C20—C25	−84.9 (10)
C1—Re1—C9—C10	173.2 (6)	C16—N1—C20—C25	95.9 (10)
C2—Re1—C9—C10	−97.2 (6)	C25—C20—C21—C22	0.1 (12)
C4—Re1—C9—C10	81.5 (6)	N1—C20—C21—C22	−179.3 (7)
C18—C9—C10—O14	1.0 (10)	C25—C20—C21—C26	179.0 (9)
Re1—C9—C10—O14	−170.3 (4)	N1—C20—C21—C26	−0.4 (12)
C18—C9—C10—C11	−177.6 (8)	C20—C21—C22—C23	0.0 (14)
Re1—C9—C10—C11	11.1 (10)	C26—C21—C22—C23	−178.9 (10)
O14—C10—C11—C12	0.6 (7)	C21—C22—C23—C24	0.1 (16)
C9—C10—C11—C12	179.2 (7)	C21—C22—C23—C27	178.1 (11)
O14—C10—C11—Re2	175.6 (4)	C22—C23—C24—C25	−0.3 (17)
C9—C10—C11—Re2	−5.7 (11)	C27—C23—C24—C25	−178.2 (12)
C5—Re2—C11—C10	179.8 (6)	C23—C24—C25—C20	0.4 (15)
C6—Re2—C11—C10	88.6 (6)	C23—C24—C25—C28	−177.4 (10)
C8—Re2—C11—C10	−89.4 (6)	C21—C20—C25—C24	−0.3 (13)
C5—Re2—C11—C12	−6.5 (7)	N1—C20—C25—C24	179.0 (8)
C6—Re2—C11—C12	−97.7 (7)	C21—C20—C25—C28	177.6 (8)
C8—Re2—C11—C12	84.3 (7)	N1—C20—C25—C28	−3.1 (12)
C10—C11—C12—C13	0.1 (8)	C15—N2—C29—C34	92.4 (10)
Re2—C11—C12—C13	−174.8 (5)	C17—N2—C29—C34	−87.7 (10)
C11—C12—C13—O14	−0.6 (8)	C15—N2—C29—C30	−89.4 (10)
C11—C12—C13—C15	174.6 (7)	C17—N2—C29—C30	90.6 (10)
C11—C10—O14—C13	−1.0 (7)	C34—C29—C30—C31	−3.7 (13)
C9—C10—O14—C13	−179.8 (6)	N2—C29—C30—C31	178.2 (7)
C12—C13—O14—C10	1.0 (7)	C34—C29—C30—C35	178.7 (9)
C15—C13—O14—C10	−175.2 (6)	N2—C29—C30—C35	0.6 (12)
C17—N2—C15—N1	0.2 (9)	C29—C30—C31—C32	2.2 (14)
C29—N2—C15—N1	−179.8 (8)	C35—C30—C31—C32	179.8 (10)
C17—N2—C15—C13	178.5 (8)	C30—C31—C32—C33	0.0 (16)
C29—N2—C15—C13	−1.5 (13)	C30—C31—C32—C36	−177.5 (10)
C16—N1—C15—N2	−0.5 (9)	C31—C32—C33—C34	−1.1 (16)
C20—N1—C15—N2	−179.8 (7)	C36—C32—C33—C34	176.4 (11)
C16—N1—C15—C13	−178.9 (8)	C30—C29—C34—C33	2.7 (13)
C20—N1—C15—C13	1.8 (12)	N2—C29—C34—C33	−179.2 (8)
C12—C13—C15—N2	−177.7 (8)	C30—C29—C34—C37	−178.3 (9)
O14—C13—C15—N2	−2.7 (11)	N2—C29—C34—C37	−0.2 (13)
C12—C13—C15—N1	0.3 (13)	C32—C33—C34—C29	−0.2 (14)
O14—C13—C15—N1	175.3 (7)	C32—C33—C34—C37	−179.1 (10)
C15—N1—C16—C17	0.7 (11)	C44—O40—C41—C42	−32.4
C20—N1—C16—C17	179.9 (8)	O40—C41—C42—C43	30.3
N1—C16—C17—N2	−0.5 (12)	C41—C42—C43—C44	−16.4
C15—N2—C17—C16	0.2 (11)	C41—O40—C44—C43	22.0
C29—N2—C17—C16	−179.8 (8)	C42—C43—C44—O40	−2.6

### r.m.s. deviations of atoms (δ_rms_, Å) and dihedral angles between planes (°) for selected mean planes

**Table:**

Plane	Atoms	δ_rms_	Plane:1	2	3	4
1	C20–C26	0.001	–			
2	C29–C34	0.011	34.8 (4)	–		
3	N1/N2/C15–C17	0.003	84.8 (3)	86.2 (3)	–	
4	C10–C13/O14	0.004	79.3 (5)	87.3 (4)	5.5 (7)	–
5	Re1/Re2/C9–C11	0.041			11.1 (6)	5.7 (4)

## References

[bb1] Bruker (2001). *SMART*, *SAINT* and *SADABS* Bruker AXS Inc., Madison, Wisconsin, USA.

[bb2] Cason, C. J. (2004). *POV-RAY for Windows* Persistence of Vision, Raytracer Pty Ltd, Victoria, Australia. URL: http://www.povray.org.

[bb3] Chia, E. Y., Naeem, S., Delaude, L., White, A. J. P. & Wilton-Ely, J. D. E. T. (2011). *Dalton Trans.* **40**, 6645–6658. 10.1039/c0dt01613f 21369614

[bb4] Farrugia, L. J. (1997). *J. Appl. Cryst.* **30**, 565.

[bb5] Kreissl, F. R., Eberl, K. & Uedelhoven, W. (1977). *Chem. Ber.* **110**, 3782–3791.

[bb6] Kreissl, F. R., Frank, A., Schubert, U., Lindner, T. L. & Huttner, G. (1976). *Angew. Chem. Int. Ed. Engl.* **15**, 632–633.

[bb7] Li, X., Schopf, M., Stephan, J., Kipke, J., Harms, K. & Sundermeyer, J. (2006). *Organometallics*, **25**, 528–530.

[bb8] Macrae, C. F., Bruno, I. J., Chisholm, J. A., Edgington, P. R., McCabe, P., Pidcock, E., Rodriguez-Monge, L., Taylor, R., van de Streek, J. & Wood, P. A. (2008). *J. Appl. Cryst.* **41**, 466–470.

[bb9] Naeem, S., Delaude, L., White, A. J. P. & Wilton-Ely, J. D. E. T. (2010). *Inorg. Chem.* **49**, 1784–1793. 10.1021/ic9021504 20088565

[bb10] Sheldrick, G. M. (2008). *Acta Cryst.* A**64**, 112–122. 10.1107/S0108767307043930 18156677

[bb11] Spek, A. L. (2009). *Acta Cryst.* D**65**, 148–155. 10.1107/S090744490804362X PMC2631630 19171970

